# Probing Membrane Structure of Lipid Nanomedicines Using Solution Small-Angle X-Ray Scattering: Applications and Prospects

**DOI:** 10.3390/membranes15120382

**Published:** 2025-12-16

**Authors:** Ke-Meng Li, Panqi Song, Xiao-Peng He, Na Li

**Affiliations:** 1 Key Laboratory for Advanced Materials and Joint International Research Laboratory of Precision Chemistry and Molecular Engineering, Feringa Nobel Prize Scientist Joint Research Center, School of Chemistry and Molecular Engineering, East China University of Science and Technology, 130 Meilong Rd, Shanghai 200237, China; likemeng2024@sari.ac.cn; 2 National Facility for Protein Science Shanghai, Shanghai Advanced Research Institute, Chinese Academy of Sciences, Shanghai 201210, China; songpq@sari.ac.cn; 3 National Center for Liver Cancer, The International Cooperation Laboratory on Signal Transduction, Eastern Hepatobiliary Surgery Hospital, Shanghai 200438, China

**Keywords:** solution small-angle X-ray scattering, lipid nanomedicine, structural characterization, AI-guided drug screening

## Abstract

Lipid-based nanomedicines are already widely used in antitumor therapy and gene delivery. However, their complex structural features demand advanced mesoscopic structural characterization tools for effective research and development (R&D) and quality control. Synchrotron small-angle X-ray scattering (SAXS) is a powerful, non-invasive technique for probing nanoscale membrane organizations, monitoring in situ dynamic membrane assembly, and exploring the interactions of components in lipid-based drug delivery systems, including liposomes, lipoplexes, lipid nanoparticles (LNPs), and lyotropic liquid crystals (LLCs). Recent advances in high-flux synchrotron facilities, high-frequency detectors, and automated SAXS data processing pipelines permit a detailed structural characterization of lamellarity, bilayer spacing, internal phases, core–shell morphology, as well as “pump-probe” dynamic process studies for lipid nanomedicines. Though major challenges remain in sample polydispersity and model fitting, the advances in time-resolved synchrotron SAXS, high-throughput automation, and artificial intelligence (AI)-assisted modeling are rapidly reducing this barrier. This review summarizes SAXS methodology and introduces representative case studies in the field of lipid nanomedicines. The performance of BioSAXS beamline BL19U2 in the Shanghai synchrotron radiation facility (SSRF) and prospects of AI-guided drug screening at BL19U2 are highlighted to advance intelligent R&D and quality control for lipid nanomedicines.

## 1. Introduction

In recent years, research on lipid-based nanomedicine delivery systems has been continuously intensified, focusing on overcoming systemic and intracellular barriers, achieving targeted and controlled drug release systems, and advancing more effective gene therapy technologies [1,2].

Lipids are fundamental components of living organisms; consequently, lipid-based nanomedicine delivery systems possess excellent biocompatibility, low immunogenicity, and high safety. Among the emerging novel nanomedicine carriers, lipid-based delivery systems—represented by liposomes and lipid nanoparticles—have been successfully commercialized and are now widely applied in antitumor therapy, the treatment of viral or fungal infections, gene editing, and other biomedical fields [2,3].

By integrating specific administration routes with desired targeting sites and rationally regulating the physicochemical properties of lipid delivery systems, precise synthesis and functional design of lipid nanomedicines can be achieved [4,5,6]. Compared with conventional pharmaceutical formulations, lipid nanomedicines exhibit unique nanoscale size, hierarchical structure, and tunable surface properties, endowing them with advantages in enhancing drug solubility, improving drug stability (both in vitro and in vivo), and boosting therapeutic efficacy [7]. Given the compositional and structural complexity of lipid nanomedicine formulations—characterized by multi-component interactions and nested hierarchical structures—traditional analytical frameworks require novel breakthroughs [8]. Therefore, it is crucial to establish a comprehensive characterization technique, which is capable of capturing nanoscale effects and dynamic structural changes, and constructing a quantitative “composition–structure–property–function” correlation model. Such a framework is of great significance for guiding the R&D as well as quality control of lipid nanomedicines.

Small-angle X-ray scattering (SAXS) is an effective technique for characterizing the nanoscale and mesoscale microstructures, enabling the acquisition of global structural parameters and characteristic information over a broad spatial resolution ranging from nanometers to micrometers [9]. Over the past few decades, with the continuous advances in experimental instrumentation and data processing methods—particularly the fast emergence of high-brilliance synchrotron radiation light sources—SAXS has been widely applied across diverse research fields, such as materials science and structural biology [10,11,12,13].

Lipid nanomedicines are formed through the self-assembly of multiple components —typically phospholipids and their structural analogs (e.g., cholesterol, ionizable lipids) —that assemble into nanoparticles with distinct structural features. This process is driven by a combination of hydrophobic, electrostatic, and van der Waals forces [14]. SAXS provides an effective means to probe the self-assembled structural characteristics of such systems, elucidating component organization and interaction mechanisms between lipid matrices and active pharmaceutical ingredients [15]. Moreover, by leveraging the high flux and temporal resolution of synchrotron light sources, SAXS enables in situ structural characterization of lipid nanomedicines under dynamic environmental changes and real-time observation of the spatial and temporal evolution of structural transitions [16,17].

With the growing understanding of the “structure–function” relationships in nanomedicines and advances in analytical techniques, regulatory agencies have increasingly recognized the importance of SAXS as an evaluation tool for critical quality control of nanomedicines. Notably, the *Technical Guidelines for Quality Control Research of Nanomedicines* issued by the Center for Drug Evaluation of the National Medical Products Administration (NMPA) explicitly identify this [18].

Building upon recent developments, this review focuses on the structural characteristics and functional response mechanisms of lipid nanomedicine systems. It systematically elucidates the fundamental principles and application strategies of the SAXS technique, highlights the technical strengths of the first synchrotron-based biological solution SAXS beamline (BL19U2) in China, and introduces its innovative methodological system developed for fine structural analysis and quality evaluation of lipid nanomedicines over the years [19,20,21,22,23,24]. In addition, representative user achievements are summarized to demonstrate the advantages of SAXS in structural characterization for lipid nano-delivery systems. Overall, this review aims to provide an innovative framework for constructing a full-industry-chain quality control standard system. In the era of AI, by integrating structural characterization, performance prediction, and producing process optimization, this framework has the potential to utilize SAXS as the core analytical platform, augmented with AI to facilitate the intelligent design and high-throughput screening of nanomedicines.

## 2. Principles and Analysis Methods of SAXS for Lipid Nanomedicines

### 2.1. Basics of Small-Angle X-Ray Scattering

X-ray scattering refers to the phenomenon where X-ray photons deviate from their original propagation direction after colliding with bound electrons outside atomic nuclei. In essence, it is an electromagnetic interaction between X-rays and the electron clouds of matter. If a substance contains uniformly distributed electrons, the photon scattering should also be spatially uniform. However, if the electron density distribution in the substance shows spatial periodicity, the scattered X-ray photons will reflect this regularity, revealing characteristic features of the measured internal structure. By tracking the X-ray scattering behavior and analyzing its characteristics, information about the internal electron density distribution within a substance can be revealed, making X-ray scattering an essential technique for analyzing material structures [25].

**Figure 1:**
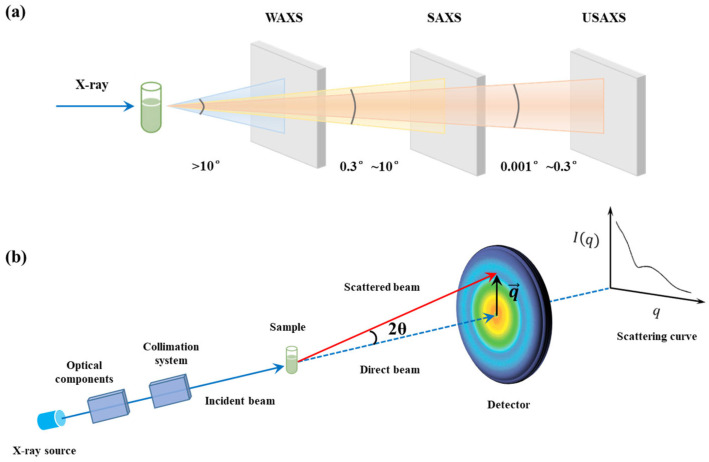
(**a**) Schematic diagram of the differences between SAXS and WAXS. (**b**) Schematic diagram of the SAXS device and technical principle.

X-ray scattering is typically classified into wide-angle X-ray scattering (WAXS, also referred to as X-ray diffraction), small-angle X-ray scattering (SAXS), and ultra-small-angle X-ray scattering (USAXS). As shown in Figure 1a, the scattering signal can be measured by adjusting the distance between the detector and the sample, altering the detection angle. SAXS typically refers to the scattering that occurs at small angles (q range typically from 0.08 nm^−1^ to 4.5 nm^−1^), while WAXS involves higher-angle scattering (q range typically exceeds 4.5 nm^−1^), and USAXS is related to ultra-low scattering angle (q range typically from 0.001 nm^−1^ to 0.08 nm^−1^). For solutions, the scattering is isotropic, and the scattered intensity depends only on the modulus of the momentum transfer q. Where with an incident X-ray wavelength λ, the magnitude of the scattering vector *q* depends on the scattering angle *θ* (the half-angle formed by the incident beam and the scattered (or diffracted) beam at the sample) [26], as described by Equation (1):

(1) $$q=\frac{4\pi \sin\theta }{\lambda }$$

Since X-ray scattering follows the reciprocal law, that is, larger real-space scales *d* correspond to smaller scattering angles, which can be derived from Bragg’s law as shown in Equation (2) [27]:

(2) $$q=\frac{2\pi }{d}$$

Therefore, scattering vector signals at different magnitudes reflect information from different spatial scales. While WAXS provides atomic-scale structural insights, SAXS enables detection from 1 nm to 100 nm, and USAXS is more suitable for detecting larger structural features up to micrometers [28]. When a sample contains structures ranging from several nanometers to hundreds of nanometers or even micrometers, coherent scattering occurs due to phase differences between scattered waves from different atoms, and this scattering signal appears within a small angular range around the incident X-ray beam. By capturing this scattering signal with a detector, the spatial distribution of the scattered X-ray photons can be recorded (as shown in Figure 1b).

For a dispersed system with randomly oriented nanoparticles, the scattering pattern is isotropic, and a one-dimensional scattering intensity profile I(q) can be obtained by performing a radial average of the two-dimensional detector pattern. The low-*q* region of the profile reflects information about the larger-scale structures, such as the size and shape of lipid nanoparticles, while the high-*q* region provides insights into smaller-scale features, such as the lipid organizations in bilayers. Structural information at different scales can be described via the relationship between scattering intensity and the *q* value in the I(q) profile, which is expressed as follows [20]:

(3) $$I\left(q\right)=n_{p}P(q)S(q)$$

In the equation, $n_{p}$ represents the number of particles per unit volume, P(q) is the form factor, and S(q) is the structure factor. The scattering intensity I(q) is a combined result of the P(q), which describes the scattering from individual particles, and the S(q), which reflects the interactions between particles. Analyzing both the form factor and structure factor of a nanoparticle is the key procedure for SAXS-based structural characterization.

When X-rays act on a single particle, the scattering intensity is determined solely by its form factor, which encodes structural characteristics such as the size, shape, and surface. In a monodispersed, unlimited diluted system, where the particle has no interactions, S(q) approximately equals 1, and the scattering intensity is a linear superposition of the scattering from individual particles. This allows for quantitative analysis of the geometric structural characteristics and particle size distributions. However, in polydisperse systems with size variations or anisotropic particles, the scattering contributions from both form factor and structure factor must be taken into consideration, for they can significantly modify the apparent structure factor S′(q) observed in experiments, leading to damped oscillations or distorted peak positions compared to the true structure factor of monodisperse systems. Three key approximations have been proposed: Kotlarchyk and Chen [29] developed the decoupling approximation (DA) for systems with modest polydispersity and anisotropy; Pedersen [30] proposed the local monodisperse approximation (LMA) for highly polydisperse systems, treating the system as a superposition of non-interacting monodisperse subsystems to enable linear fitting of size distributions; and the scaling approximation (SA) leverages corresponding states theory to scale parameters from monodisperse reference fluids, accurately capturing excluded volume effects and outperforming DA/LMA in reproducing structure factor behavior for hard-sphere and Lennard–Jones systems [31]. These methods collectively enable quantitative analysis of particle size distributions, spatial correlations, and interaction potentials in complex colloidal, micellar, or nanoparticle systems by integrating form factor and structure factor contributions, providing additional insights into the spatial correlations between particles and their interaction potentials.

### 2.2. Scattering Data Processing and Model Fitting

Accurate SAXS data analysis begins with proper data pre-processing, including background subtraction, intensity normalization, and absolute scaling. Background subtraction removes background scattering contributions from buffers, solvents, and sample holders, isolating the intrinsic scattering signal of the nanoparticles [32,33]. Absolute intensity calibration, often performed using water or glassy carbon standards, enables conversion of relative intensity into absolute units (cm^−1^), allowing direct quantitative evaluation of measured systems. It should be noted that the absolute scaling is critical for the quantitative determination of structural parameters such as molecular mass, particle volume fraction, or electron-density contrast [34].

Typically, 1-dimensional (1D) structural parameters can be directly calculated from the scattering profile without assuming a specific shape. In the Guinier regime (low-*q*), the scattering profile can be approximated as $I\left(q\right)=I\left(0\right)exp\left(-q^{2}R_{g}^{2/3}\right)$ [35], providing the calculated value of radius of gyration (R_g_) and the forward scattering intensity I(0), which are indicative of overall particle size and molecular weight of measured nanomedicines. Moreover, at high-*q* range, the so-called Porod regime indicates that I(q) proportional to *q*^−4^, from which the specific surface area and inter-facial roughness can be extracted. These analyses offer rapid insights into particle size, compactness, and surface properties, providing the fundamentals for further model fitting.

For lipid-based assemblies, such as liposomes, LNPs, and LLCs, the scattering intensity can be described by the product of a form factor P(q) and structure factor $S\left(q\right)$ (Equation (3)). Here, P(q) reflects the internal electron-density distribution of lipid bilayer thickness, core–shell contrast, or multilamellar order, while S(q) represents interparticle correlations, packing interactions, and long-range mesophase ordering [15,36]. Typical SAXS patterns of lipid assemblies display characteristic lamellar Bragg peaks, corresponding to the periodic repeat distance $d=\frac{2\pi }{q}\;$ [37], low-angle peaks associated with cubic (e.g., La3d, Pn3m) [16] or hexagonal (H_II_) mesophases [38,39], and broad features indicative of polydispersity or structural disorder. Approaches based on the Generalized Indirect Fourier Transformation (GIFT) method and relying on the real space representation of the form factor by the distance distribution and of the structure factor by the pair correlation function were also proposed [40,41]. Quantitative fitting using multilayered-sphere, core–shell, or Gaussian electron-density models allows determination of membrane thickness, internal spacing, and hydration profiles essential for understanding encapsulation behavior and membrane mechanics [42,43].

Beyond traditional form-factor or multilayer electron-density models, DENSS (Density from Solution Scattering) provides a powerful ab initio approach for reconstructing three-dimensional (3D) electron-density maps directly from solution scattering data. Originally developed by Grant et al., DENSS operates by iteratively refining a 3D density distribution whose calculated scattering profile best matches the experimentally measured SAXS/SANS curve [44]. Unlike real-space modeling methods based on bead approximations (e.g., DAMMIN, DAMMIF) [37,45], DENSS reconstructs a continuous volumetric density, making it particularly well suited for soft-matter and lipid-based assemblies whose internal structures are defined by smoothly varying electron-density gradients. In the context of liposomes, LNPs, and lyotropic liquid crystalline mesophases, DENSS enables visualization of features that are difficult to capture with parametric form-factor models, such as bilayer asymmetry and curvature-induced density variations, internal water-channel networks in cubic or hexagonal phases, heterogeneous core–shell organization in LNPs, including regions enriched in nucleic acids or ionizable lipids, and partial distribution of PEG layers, hydration shells, or encapsulated drugs [46,47]. Given the intrinsic complexity and dynamic heterogeneity of lipid nanomedicines, SAXS analysis increasingly benefits from hybrid modeling strategies that combine information from complementary techniques. In detail, the small-angle neutron scattering (SANS) provides contrast variation through isotope labeling, enabling decoupling of different components within multi-layered systems [48,49,50]. Cryo-electron microscopy (cryo-EM) offers high-resolution morphological validation, providing information for mathematical model establishment [51,52,53]. Molecular dynamics (MD) simulations can provide atomic-level structural fluctuations and enable SAXS profile theoretical calculation for further model validation [16,17]. Notably, asymmetrical-flow field-flow fractionation (AF4) coupling with SAXS offers a novel approach for obtaining quantitative and size-dependent information on nano-pharmaceuticals and colloids [54,55]. Integration of SAXS with these complementary tools enables hybrid models that reconcile experimental scattering data with physically realistic 3-dimensional (3D) structural ensembles. De Mel et al. [56] investigated the interaction between acetaminophen (APAP) and large unilamellar vesicles (LUV) composed of DOPC by analyzing the scattering data with a spherical shell model. Schilt et al. [57] determined the thickness and density of the PEG layers and the structure of the drug inside the liposomes based on a spherical model.

## 3. Typical Applications of SAXS in Lipid-Based Nanomedicine Characterization

### 3.1. Liposomes

**Figure 2:**
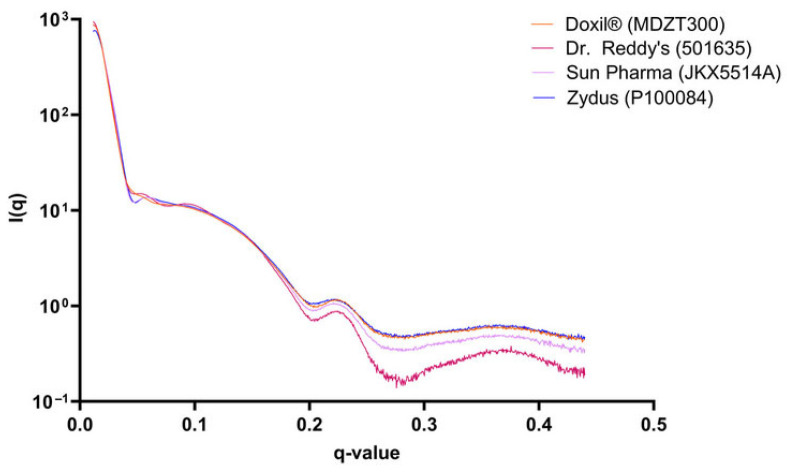
SAXS profiles for one liposomal Doxil formulation representative of each manufacturer [59].

Liposomes are spherical vesicles consisting of curved lipid bilayers and hydrophilic cavities, and have been considered as a promising carrier for a variety of therapeutic agents, such as small molecule drugs and genes [58]. In industrial research on liposomes, the SAXS technique has emerged as a critical investigative tool. Owing to its precise ability to resolve nanostructures, SAXS excels at capturing subtle differences in internal lamellar organization. Eneli et al. [59] compared the innovator liposomal drug Doxil^®^ (Baxter Healthcare, Deerfield, IL, USA) with generic products (Dr. Reddy’s, Hyderabad, Telangana, India; Sun Pharmaceuticals, Goregaon, Mumbai, India; Zydus, Ahmedabad, Gujarat, India). While cryo-TEM observed a similar “coffee-bean” morphology and thread-like doxorubicin-sulfate crystals in the aqueous core across all samples, SAXS further revealed distinct nanostructural differences in their membrane and mesoscopic structures: Dr. Reddy’s generic showed scattering signals consistent with more uniform lamellar periodicity, corresponding to its higher liposome uniformity observed via cryo-TEM, whereas Sun Pharma and Zydus generics exhibited heterogeneous profiles, aligning with their relatively lower structural consistency. These SAXS-derived differences could explain variations in the state of encapsulated drug, as nanostructural periodicity directly impacts drug–lipid interactions and encapsulation stability. Notably, despite these structural disparities, all formulations showed comparable in vitro drug release, yet the SAXS findings highlight that surface-level morphological similarity does not equate to nanostructural equivalence (Q3 compliance). Such discrepancies, rooted in subtle manufacturing process deviations, lipid purity fluctuations, and structural homogeneity variations, carry real-world implications for generic bioequivalence evaluation, process optimization, and quality control of complex liposomal formulations—ensuring clinical safety and efficacy beyond basic in vitro release metrics (Figure 2).

In addition, Maeki et al. [60] used a microfluidics-based time-resolved SAXS system to track ethanol-induced lipid bilayer changes from uni- to multilamellar. The technique captured dynamic phase-related structural shifts, highlighting that residual ethanol disrupts the assembly of liposomes—underscoring the critical need for ethanol removal in liposome manufacturing and demonstrating SAXS’s value in studying dynamic phase behaviors.

### 3.2. Lipoplexes

Lipoplexes represent a fundamental class of delivery vehicles formed spontaneously through the electrostatic interactions between cationic lipids and negatively charged nucleic acids. The nucleic acids are either encapsulated within the lipid layers or adsorbed onto the lipid surface, resulting in aggregate-like structures [61,62]. However, characterizing these complexes presents significant challenges. Unlike the relatively uniform and predictable morphologies of liposomes, lipoplexes are characterized by substantial structural irregularity. The size of these aggregates and the arrangement of lipid membranes are highly susceptible to formulation variables, particularly the lipid types and nucleic acid loading ratio, leading to high polydispersity that resists precise characterization via standard biophysical techniques.

**Figure 3:**
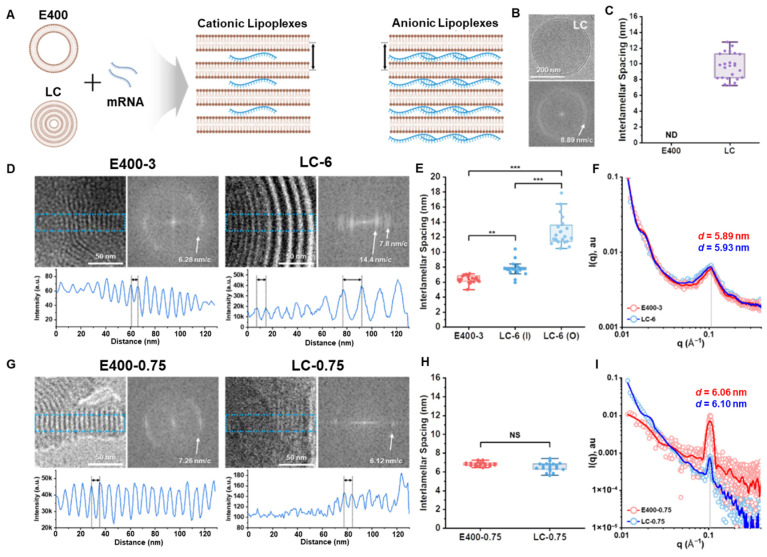
Comparison of self-assembly structures of E400 and LC-based lipoplexes. (**A**) Schematic representations of unilamellar E400 and multilamellar LC with the ordered structure of the corresponding cationic and anionic lipoplexes, highlighting interlamellar spacing. (**B**) Representative cryo-EM and FFT images of LC revealing a frequency of 8.89 nm/c (bilayer repeat). (**C**) Interlamellar spacings of E400 and LC analyzed using the pixel intensity profile from cryo-EM images (*n* > 20 for each sample, mean ± S.D.). (**D**) Representative cryo-EM images of cationic lipoplexes highlighting the ordered structure and corresponding FFT images and pixel intensity profile to analyze the repeat distances. (**E**) Interlamellar spacing of cationic lipoplexes analyzed from cryo-EM images (*n* > 20 for each sample). I and O denote the inner and outer parts of LC-6, respectively. (**F**) SAXS profiles for cationic lipoplexes with d-spacing calculated from the peak position. (**G**) Representative cryo-EM images of anionic lipoplexes highlighting the ordered structure and corresponding FFT images and pixel intensity profile to analyze the repeat distances. (**H**) Interlamellar spacing of anionic lipoplexes analyzed from cryo-EM images (*n* > 20 for each sample). (**I**) SAXS profiles for anionic lipoplexes with d-spacing were calculated from the peak position. Data are reported as the mean ± standard deviation, and *p* values of <0.05 were considered statistically significant (** *p* < 0.01, and *** *p* < 0.001) [42].

Due to its high-resolution capability for nanoscale structural analysis, SAXS overcomes these limitations. Uniquely suited for disordered systems, SAXS enables the bulk characterization of key parameters for lipoplexes, including interlamellar spacing and phase distribution. For instance, SAXS has been successfully employed to determine the lamellar structure of lipoplexes and calculate the interlamellar spacing of them (Figure 3), providing an intriguing insight into the relationship between lipid assembly structures and the biophysical characteristics of the resulting lipoplexes [42]. Based on the lamellar structural features of lipoplexes revealed by SAXS, Settanni et al. [17] utilized MD simulations to further elucidate the dynamics of DODMA-based lipoplexes for mRNA delivery, demonstrating the potential of lipoplexes in drug delivery. To further optimize lipoplex systems, neutral phospholipids or PEGylated lipids are often added to the formula to enhance structural stability, leading to the development of lipid nanoparticles (LNPs). The clinical relevance of such lipid-based assemblies is underscored by regulatory milestones, such as the FDA approval of lipid complexes like Abelcet, which demonstrates the therapeutic viability of these systems. This structural refinement has been instrumental in the evolution from simple lipoplexes to sophisticated LNPs.

### 3.3. Lipid Nanoparticles (LNPs)

**Figure 4:**
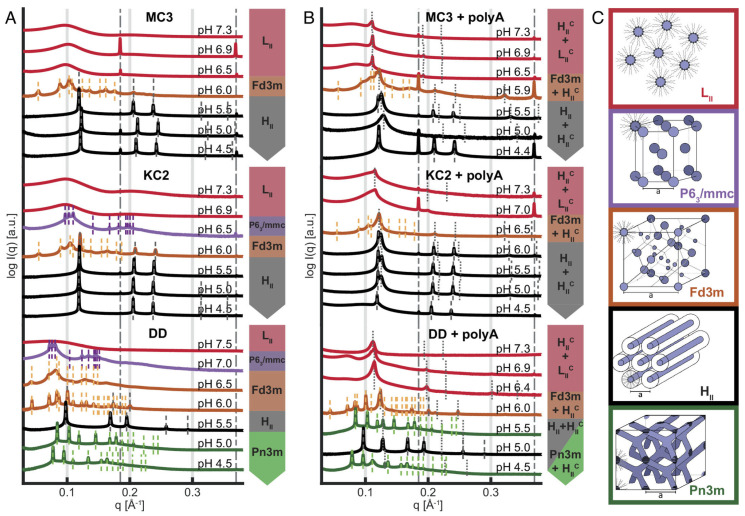
SAXS−based identification of pH−dependent mesophase transitions. (**A**) Ionizable lipid, chol, and buffer, and (**B**) ionizable lipid, chol, buffer, and polyA. Data show the three ionizable lipids, MC3, KC2, and DD, for comparison. (**C**) Schematic representation of lipid phases with decreasing pH from top to bottom, showing a trend from negative curvature toward zero curvature with increasing headgroup protonation: inverse micellar fluid isotropic L_2_, inverse micellar cubic with P63/mmc symmetry, inverse micellar cubic with Fd3m symmetry, inverse hexagonal H_2_, and bicontinuous cubic Pn3m. In the presence of polyA (**B**), the coexistence of lipid mesophases with a complexed, nucleic acid-containing phase is observed. In the pH range from 5.0 to 6.0, typically, H_2_ and H_2_^c^ coexist [16].

Lipid nanoparticles (LNPs) are regarded as a specialized form of liposomes, composed of four key components: a cholesterol, a neutral phospholipid, a PEGylated lipid, and—most importantly—an ionizable cationic lipid (CIL) [63,64]. The distinctive property of CILs is their ability to acquire a positive charge under acidic conditions, enabling strong electrostatic interactions with negatively charged nucleic acids to form dense core–shell nanostructures. As a result, LNPs have been widely adopted for nucleic acid delivery, and SAXS has played an indispensable role in analyzing their structural parameters and the crystalline structures [43,65]. In the study by Philipp et al. [16], SAXS is used to monitor Bragg peak changes in mRNA-LNPs with three CILs as pH decreased from 7 to 5. By analyzing the *q*-value ratios of multiple peaks, they identified sequential phase transitions: from disordered inverse micellar (L_2_) to inverse micellar cubic (Fd3m), and, finally, to inverse hexagonal (H_2_) phases.(Figure 4) These pH-dependent rearrangements directly explained differences in nucleic acid release efficiency, providing a framework to optimize CIL formulations for intelligent drug delivery. SAXS also provides direct evidence for multi-shell or disordered core–shell architectures of LNPs.

**Figure 5:**
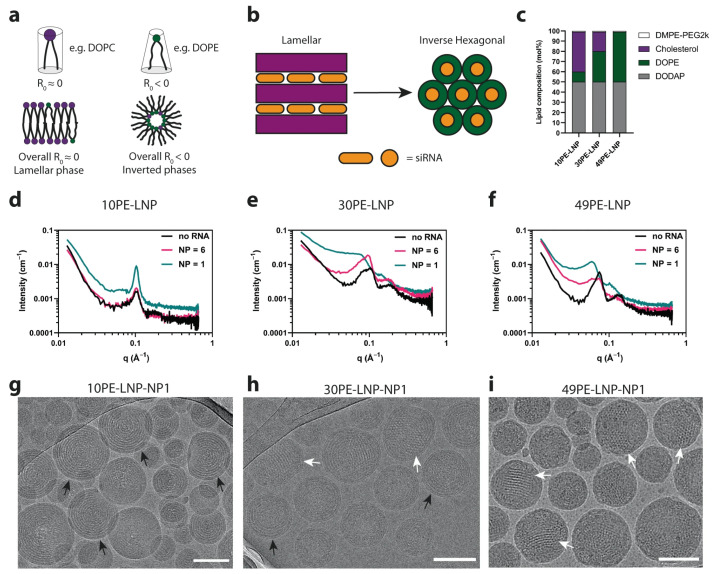
(**a**) Curvature in lipid mixtures is driven by the composition of lipids with various curvature profiles. Compositions with larger amounts of lipids with an R_0_  <  0 lead to the formation of inverted phases. (**b**) Lamellar structures can transition towards inverted phases with the increase in DOPE lipid content. (**c**) LNP compositions designed to form lamellar and inverted lipid structures encapsulating siRNA used in this study. (**d**–**f**) SAXS profiles of the LNP compositions shown in c, at different N/P ratios. (**g**–**i**) Representative cryo-TEM images of the LNPs compositions in c, at NP ratio = 1. Scale bars are 100 nm. Black arrows indicate the presence of lamellar structures. White arrows indicate the presence of nonlamellar structures [68].

Gilbert et al. [66] combined SAXS with SANS, dynamic light scattering (DLS), and cryo-TEM to study cargo (DNA, polyA, polyU) and N/P ratio effects on LNPs lipid distributions. SAXS data fitting revealed that both loaded and empty LNPs contributed to scattering signals, with loaded-particle signals diminishing at higher N/P ratios in SANS profiles—indicating more efficient nucleic acid encapsulation and denser core–shell packing. For clinically approved siRNA-LNPs like Onpattro^®^ (Patisiran) [67], SAXS further clarifies how formulation directly influences the membrane structure, laying the groundwork for structural optimization. Pattipeiluhu et al. [68] designed three siRNA-LNP formulations with varying DOPE proportions and used SAXS to characterize structural differences. As shown in Figure 5, SAXS profiles showed that with different content of DOPE, LNPs exhibit different structures: 10PE-LNP profiles displayed a Bragg reflection at a scattering vector *q*∼0.1 Å^−1^, and the associated cryo-TEM images exhibited the formed concentric circles extending to the LNP core, revealing the formulation of a lamellar (Lα) phase. When the DOPE content was increased to 30 mol% (30PE-LNP), the Bragg reflection shifted towards smaller *q* values and broadened. Associated with cryo-TEM images, it is indicated that mixed lamellar and inverse hexagonal phases (H_II_) were formed. And 49PE-LNP exhibits further shifted *q* values consistent with sole H_2_ phases (i), where variations in form factors directly mirror differences in lipid periodicity and arrangement. These subsequent delivery studies linked phase order to efficiency: Lα phases often supported more stable siRNA encapsulation, while H_2_ phases facilitated siRNA release. This SAXS-derived structure–performance link is pivotal for optimizing LNPs for nucleic acid delivery.

### 3.4. Lyotropic Liquid Crystals (LLCs)

Lyotropic liquid crystals (LLCs) are highly ordered, self-assembled supramolecular intermediates formed by the association of amphiphilic lipid molecules into micellar aggregates in polar solvents, typically in water [69]. These systems exhibit highly ordered nanostructures and thermodynamic stability, making them exceptionally promising candidates for controlled and sustained drug release applications. LLCs can adopt various mesophases such as lamellar, hexagonal, cubic, and sponge phases, which critically influence their drug-loading capacity, release behaviors, and bioavailability [70,71,72].

The specific mesophase adopted by LLCs is highly dependent on factors like lipid concentration, temperature, and hydration. Therefore, understanding the kinetics of drug release and the drug loading process is critical for the biocapacity LLC’s drugs. Synchrotron SAXS is a non-destructive technique and can be applied in dynamic monitoring of structural evolution in response to external stimuli, making it uniquely suited for characterizing the repeating lattice and liquid crystalline phase of LLCs.

Liu et al. [73] investigated a temperature-sensitive LLCs system for transdermal drug delivery. Using SAXS in combination with rheology and low-field nuclear magnetic resonance (LF-NMR), they demonstrated a temperature-dependent phase transition from cubic to hexagonal and lastly to micellar solution in a Tween 80/RH 60/EtOL/water system. The incorporation of paeonol was found to modulate the transition temperature closer to the physiological range, enabling temperature-triggered drug release. In this study, the key structural changes observed via SAXS included the successive transition from a highly ordered cubic mesophase to a hexagonal phase, culminating in a disordered micellar solution upon heating. Crucially, the incorporation of the therapeutic agent, paeonol, was found to modulate the transition temperature, shifting it closer to the physiological range. This structural manipulation enabled the system to be engineered for temperature-triggered drug release, providing strong experimental evidence for the design of intelligent, stimuli-responsive drug delivery systems.

## 4. Case Study: NFPSS BL19U2 Beamline in SSRF

**Figure 6:**
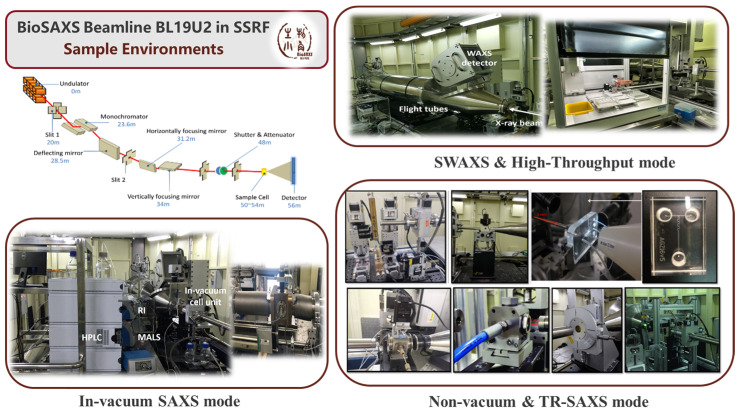
Schematic diagram of experimental setups of BL19U2 beamline.

BL19U2 beamline, operated by the National Facility for Protein Science Shanghai (NFPSS) at Shanghai Synchrotron Radiation Facility (SSRF), represents China’s first dedicated SAXS beamline specifically designed for the structural characterization of biological macromolecules and soft-matter systems in solution (Figure 6). While originally optimized for biological macromolecules, BL19U2 has evolved into one of the most versatile sample environments SAXS platforms for probing lipid-based nanomedicines, liquid crystalline nanostructures, and colloidal assemblies in situ, under physiologically relevant conditions. Its experimental configurations enable high-throughput measurements, time-resolved studies, and quantitative model reconstruction, making it a representative of how synchrotron SAXS facilitates current nanomedicine research.

### 4.1. Experimental Setups of BL19U2

#### 4.1.1. SAXS Mode with Non-Vacuum Pipes

The non-vacuum SAXS mode is the standard operational configuration for solution scattering experiments at BL19U2. In this setup, the sample cell is positioned in air [21]. This setup allows for rapid sample exchange with automated flow cells with a flexible tuning of the scattering vector q. The sample-to-detector distance can be adjusted from 1 to 7 m (typically fixed at ~2.6 m for BioSAXS) and is compatible with temperature, pH, and other sample environment changes.

In lipid nanomedicine research, this setup enables real-time monitoring of self-assembly behavior, structural evolution, and stability of LNPs, liposomes, and lipoplexes under solution conditions. Combined with temperature-controlled sample environments and automated flow cells, BL19U2 supports dynamic measurements such as drug–lipid interactions, phase transitions, and pH- or ion-induced structural changes, offering valuable insights into the formulation stability and delivery performance of lipid-based systems. Benefited from the good signal-to-noise performance of BL19U2, the in situ phase transition of oil/surfactant/water systems was measured for a novel lipid-based drug delivery system of antitumor drug bufalin [74]. The SAXS patterns demonstrated specific structural organizations within the reported formulation. In the process of water dilution, the emergence of first and second-order Bragg peaks indicated the formation of lamellar structures or multilamellar nanoparticles. By analyzing the q-vector values of these peaks, specific interlayer repeat distances (*d*-spacing) were calculated as 14.9 nm. In addition, SAXS was critical in evaluating the encapsulation of bufalin. The scattering curves revealed that while the internal liquid crystalline structure was preserved after drug loading, the Bragg peaks shifted toward smaller q. This shift indicated an expansion in interlayer spacing, providing structural evidence that the drug molecules were successfully intercalated into the surfactant membrane reservoirs. Ultimately, the SAXS data established the structural basis for the system’s viscosity changes and sustained drug release profiles.

#### 4.1.2. SAXS Mode with Vacuum Pipes

The in-vacuum configuration in BL19U2 improves data quality by eliminating air scattering and reducing background noise [20]. This configuration significantly improves scattering data quality at ultra-low *q* values—critical for structural characterization of long-range lamellar periodicity in multilamellar vesicles, inter-layer spacing in LLC mesophases, and aggregates or fusion intermediates of LNPs [48,75].

The vacuum configuration is particularly suited for quantitative electron-density profiling and precise model fitting of bilayer assemblies, such as resolving the bilayer thickness, distance of inter spacing, hydration shell thickness, and phase symmetry. Complementing this focus on data quality, the BL19U2 beamline also integrates size exclusion chromatography (SEC)–SAXS coupling technique to ensure sample homogeneity. The SEC-SAXS setup combines an Agilent 1260 Infinity HPLC, Wyatt MALLS/DLS detectors, and an X-ray exposure cell [19]. Sun and his group benefited from this in-line in vacuum SEC-SAXS setup to successfully characterize the case in the dissociation process in a polydisperse surfactant–protein system [76]. The high-brilliance X-rays were used to monitor the structural evolution of caseinate particles and casein micelles when exposed to surfactants like SDS, CTAB, and polysorbates. The collected scattering data were fitted with theoretical models (e.g., decorated core–shell model, beads-on-string model, etc.), and the specific conformation changes, such as unfolding process, formation of surfactant–protein complexes, and the transition of beta−casein into alpha−helical under particular surfactant concentrations were well characterized.

#### 4.1.3. SWAXS Mode

The SWAXS mode integrates small-angle (SAXS) and wide-angle (WAXS) scattering in a single experimental setup, enabling multiscale structural characterization from the nanometer to atomic range [20,21]. This configuration is critical for lipid nanomedicine research, where nanoscale mesoscopic order (e.g., lamellar spacing, vesicle size, or cubic phases) and molecular packing (e.g., lipid tail alignment and crystallinity) can be correlated.

In studies of lipid nanomedicines, this SWAXS mode enables identification of chain ordering transitions during formulation screening and quantification of cholesterol-dependent membrane packing. It is also practical in monitoring the crystalline drug domains within nanomedicines and further correlates mesophase transitions with functional properties. Li and her group benefited from the SWAXS mode in BL19U2 and conducted time-resolved scattering measurements to elucidate the self-assembly mechanism of amphiphilic Janus heteroclusters [77]. The high-flux and wide spatial resolution allowed researchers to monitor structural changes in real-time from the atomic level up to tens of nanometers as acetone evaporated from a mixed solvent solution. The application of SWAXS was necessary to identify a three-step structural evolution from fractal, core–shell nanoclusters to the formation of superlattice crystallization, providing a molecular-level understanding of the dynamic assembly from individual building blocks to complex superlattices.

### 4.2. Applications to Lipid Nanomedicine Structural Characterization

**Figure 7:**
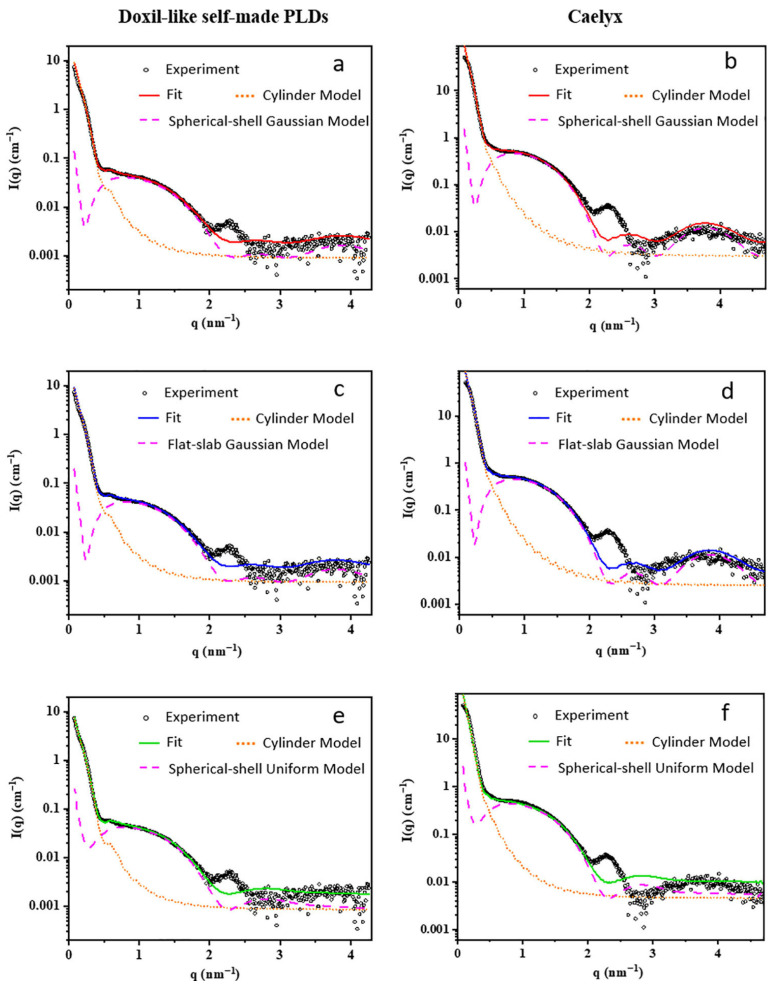
SAXS profiles of the self-made PLDs (**a**,**c**,**e**) and Caelyx (**b**,**d**,**f**). The best-fitting profiles by different analytical models are also shown. The fitting signal of PLDs (solid line) was a combination of a liposomal membrane model (dashed pink line) and a nanocrystal model (dotted orange line).

Benefited from its high brilliance and versatile sample environments, the BL19U2 beamline has become a practical analytical platform for studying the “formulation-structure–function” relationship of lipid nanomedicines. Li et al. [78] combined SR-SAXS with fitting models to characterize the internal crystal structure of Doxil-like PEGylated liposomal doxorubicin (PLD), including Caelyx and a laboratory-prepared analog. Spherical-shell/flat-slab geometries with Gaussian electron density profiles modeled the liposomal membrane, while a cylinder model fitted the encapsulated drug crystal (Figure 7). Results showed Gaussian-distribution models were suitable for membrane structure elucidation under specific scattering vectors, whereas uniform-density models exhibited poor fitting. This work highlights SAXS’s capability to provide nanoscale structural information, offering a reliable, user-friendly method to facilitate SAXS applications in nanopharmaceutical production and regulation.

**Figure 8:**
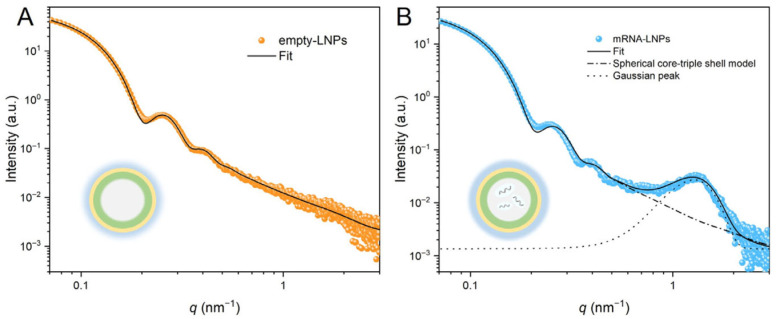
Experimental and fitted SAXS curves of LNPs: (**A**) empty LNPs, (**B**) mRNA-LNPs. The insets show schematic representations of empty LNPs and mRNA-LNPs.

The beamline supports in situ kinetic studies, such as real-time tracking of LNPs self-assembly during microfluidic mixing, drug encapsulation dynamics [48], and membrane remodeling under physiological stimulation [22]. Li et al. [79] presented a core–triple-shell SAXS model that resolves LNPs’ hierarchical organization (Figure 8), including the inner lipid layer, intermediate hydrophilic region, and outer PEG corona. For LNPs encapsulating mRNA, a Gaussian distribution model was implemented to characterize the quasi-periodic structure originating from the self-assembly of mRNA-ionizable lipid complexes. This modeling framework provides pharmaceutical researchers with robust analytical tools for systematic stability assessment and precision formulation for the optimization of LNPs. Li et al. [80] demonstrated the existence of a lamellar liquid crystal structure in the emulsion based on the ratio of peaks from SAXS curves. SAXS data collected from BL19U2 have enabled quantitative reconstruction of lamellar and non-lamellar phase transitions, particle heterogeneity, and internal electron-density distributions, offering molecular-level insights that complement imaging and spectroscopic methods.

## 5. AI-Driven SAXS Data Analysis: Challenges and Prospects

### 5.1. Challenges in Traditional SAXS Data Interpretation: Model Ambiguity, Polydispersity, Manual Fitting

While SAXS is proven as an indispensable tool for characterizing lipid nanostructure, its conventional application in the field of lipid nanomedicines still faces several challenges, leading to inefficiency and inconsistencies in structural assessment.

The primary limitation stems from model ambiguity, which arises because of complex lipid nanomedicine formulations. For example, LNPs are rarely composed of a singular, perfect phase. Instead, they often contain co-existing structures (e.g., vesicles, micelles, or internal crystalline domains) and a sheath-like outer layer [81,82]. Therefore, they normally exhibit overlapping and non-uniform structures. Accurately fitting the scattering profile with a single theoretical model becomes mathematically challenging and often physically questionable. Researchers are often forced to resort to fitting based on bulk phase structures to simplify the data analysis with a priori assumptions that may not reflect the true internal state of the LNPs in vivo, leading to over-simplification and a non-unique solution to the fitting problem [16,38].

In addition, the inherent polydispersity in size, shape, or phase distribution of lipid nanoparticles significantly compromises the quantitative reliability of SAXS data analysis [78,79]. Generally, SAXS collects an ensemble-averaged signal from millions of particles. In case a sample is highly polydisperse, the resulting scattering profile is quite complicated with a smoothed convolution of many different individual structures. This averaging effect obscures characteristic features in the scattering signal, making the precise extraction of critical structural parameters highly problematic and further resulting in the bias of quantitative assessment and batch-to-batch quality control.

Last but not least, the traditional SAXS data analysis heavily relies on manual model fitting [49,50,83,84], which is both inefficient and fundamentally subjective. It requires experienced researchers to iteratively adjust numerous model parameters based on trial-and-error and expertise, which dramatically limits the feasibility of SAXS for high-throughput screening in R&D. Crucially, the reliance on subjective judgment introduces significant variability. Fitting results for the same batch of scattering profile can vary substantially across different operators, laboratories, or even different analysis sessions, undermining the reliability and reproducibility of the data reduction.

These limitations collectively request the urgent need for integrating advanced, automated, and objective scattering data processing tools to unlock the potential of SAXS in accelerating rigorous lipid nanomedicine R&D. AI-empowered machine learning and automated high-throughput data processing pipelines should be the future.

### 5.2. AI-Assisted Analysis Pipelines

Artificial intelligence (AI) and machine learning (ML) have emerged as a transformative shift in drug discovery, particularly in streamlining the laborious screening and structural characterization of complex systems like lipid nanomedicines.

**Figure 9:**
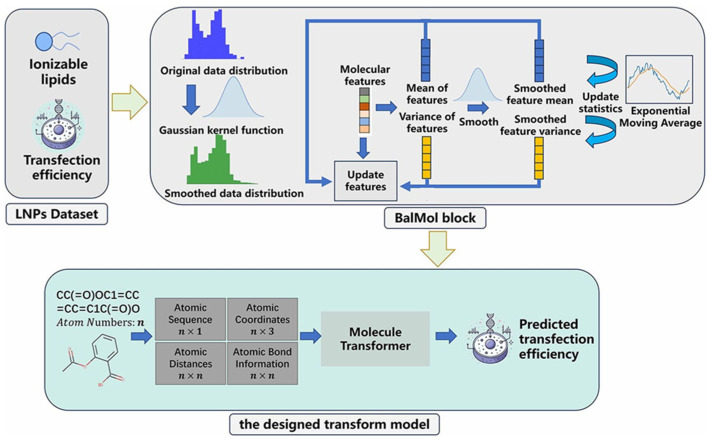
The overall architecture for predicting the properties of ionizable lipids begins with obtaining transfection efficiency datasets for various ionizable lipids. Subsequently, the BalMol block is used to smooth the distribution of labels and molecular features for balancing the data of LNPs. Finally, the TransLNP model is employed to predict transfection efficiency.

Transformer models are now being developed to predict the pharmaceutical efficiency of formulations in silico, circumventing the high cost and time required for empirical drug discovery. Wu et al. [85] address the inefficiency and high cost of traditional LNPs screening for mRNA delivery by proposing TransLNP, a transformer-based model integrated with the BalMol block (Figure 9). TransLNP leverages both coarse-grained atomic sequence and fine-grained atomic spatial relationship information, combined with pretraining via 3D coordinate reconstruction and masked atom prediction, as well as data balancing through label and molecular feature distribution smoothing to predict LNPs’ transfection efficiency. The model outperforms state-of-the-art methods under both random and scaffold data splitting, identifies 4267 molecular transfection cliff pairs as key prediction error sources, and accelerates LNPs development for mRNA drugs, providing ideas for the functional prediction of lipid nanomedicines. There are also reports on AI tools for SAXS data analysis.

AI tools can also directly address the challenges of model ambiguity and manual subjectivity in SAXS data processing. For pattern recognition, machine learning (ML) algorithms can automatically identify morphology features in scattering profiles—they learn from a labeled dataset to classify lipid nanosystem phases within seconds, eliminating manual interpretation bias [86,87,88,89]. In automated model fitting, ML models are trained on extensive datasets from both simulated and experimental scattering profiles to optimize fitting parameters and enhance the signal-to-noise ratio (SNR) [90]. This process dramatically reduces the fitting time from hours to minutes while simultaneously improving the accuracy of derived structural parameters. This data automation is critical for obtaining reliable, quantitative structural results from highly heterogeneous systems like drug-loaded liposomes and LNPs complexes. Furthermore, a promising approach for deep structural analysis is the integration of AI with molecular dynamics (MD) simulations. In this case, AI models can rapidly predict initial plausible structural models directly from raw scattering profiles [91,92]. The MD simulation then takes this AI-generated model as a starting point, refining it to simulate the precise dynamic molecular interactions over time. This synergy enables precise prediction of complex phase transition phenomena and offers a powerful feedback loop for optimization of formulation parameters.

Looking ahead, integrating AI with SAXS data represents a crucial step forward. This synergy could enhance current computational understanding of dynamic lipid nanomedicines conformations and establish robust structure–function relationships. By refining the structural models used in prediction, AI-SAXS integration is proposed to enable the more accurate, efficient, and reliable screening of next-generation drug delivery systems, moving the current field from iterative trial-and-error toward intelligent, high-precision formulation engineering.

### 5.3. Intelligent R&D and QC Framework

AI-driven SAXS has the potential to reshape current research and quality control (QC) workflows for lipid-based nanomedicines by enabling a more intelligent, highly automated, and data-centric framework. In real-time process control, SAXS-AI coupling creates a closed feedback loop. In this loop, SAXS provides high-resolution, in situ scattering profiles that capture structural evolution during manufacturing, while AI algorithms can rapidly interpret these data streams, identify deviations from target structural states, and recommend immediate adjustments to process parameters. This capability is particularly valuable for complex lipid formulations where subtle nanoscale variations have significant functional consequences [60].

Looking ahead, smart beamlines integrated with AI will further enhance throughput and reproducibility by automating key experimental steps, such as sample introduction, data acquisition, preprocessing, and preliminary model fitting. Such automation not only reduces manual bias but also enables systematic, large-scale screening of formulation variables that would be impractical with conventional workflows.

Additionally, digital twin platforms—which integrate SAXS-derived structural data, AI-predicted performance, and multi-scale biological information (in vitro/in vivo)—will establish a “nanostructure–function” correlation model. These virtual systems can learn correlations between nanostructure, process conditions, and therapeutic performance, allowing researchers to simulate formulation behavior, forecast stability or delivery efficiency, and refine design parameters before physical experiments are conducted. Critically, this approach elevates SAXS from a purely analytical tool to a central component of an iterative “design–build–test–learn” cycle, accelerating R&D and enabling precise, evidence-based QC throughout the entire product lifecycle.

## 6. Conclusions

This review systematically summarizes the current applications and technical advantages of synchrotron-based SAXS in the structural characterization of lipid-based nanomedicine delivery systems. Owing to its high-resolution nanoscale structural analysis, capability for in situ dynamic monitoring, and strong synergy with complementary non-destructive techniques like cryo-TEM, SANS, and MD simulations, SAXS has become an indispensable tool for elucidating the multi-level structures of systems such as liposomes, LNPs, lipoplexes, and LLCs. It provides unique insights into membrane organization, phase behavior, drug loading mechanisms, and structure–function relationships. In industrial manufacturing, SAXS also offers novel and solid data support for generic drug equivalence evaluation, formulation process optimization, and the design of intelligent drug delivery systems. Notably, the BL19U2 beamline of the National Facility for Protein Science in Shanghai serves as a critical technical platform, which further strengthens the practical application of SAXS technology in lipid nanomedicine research—particularly in mainland China.

With the rapid development of artificial intelligence (AI), the application of SAXS in lipid-based drug research is promising to become more intelligent and systematic. On one hand, AI can enable high-throughput data processing and modeling of SAXS datasets, using machine learning algorithms to automatically identify structural features in scattering profiles, optimize fitting models, and predict phase behavior and release kinetics. This will significantly enhance the efficiency and accuracy of data analysis, facilitating a closed-loop intelligent pipeline encompassing “structural characterization–performance prediction–process optimization”. On the other hand, the construction of a dedicated corpus for lipid-based drug delivery systems will serve as a critical foundation for AI-driven drug screening. Such a corpus should integrate multi-scale structural data from SAXS, DLS, cryo-TEM, and other techniques, and correlate them with key attributes including formulation composition, process parameters, and in vitro/in vivo performance. By leveraging AI-powered data mining and analysis, deeper structure–function relationships can be discovered, accelerating the rational design and optimization of novel lipid carriers. Ultimately, these advancements will contribute to intelligent R&D and precise quality control of lipid nanomedicines.

## Data Availability

Not applicable.
